# Large‐scale Plasma Proteomic Profiling Identifies a Robust Set of Biomarkers for Detection of Clinical Alzheimer's Disease

**DOI:** 10.1002/alz.089494

**Published:** 2025-01-09

**Authors:** Gyujin Heo, Muhammad Ali, Jigyasha Timsina, Menghan Liu, Carlos Cruchaga, Yun Ju Sung

**Affiliations:** ^1^ Washington University School of Medicine, Saint Louis, MO USA; ^2^ Department of Psychiatry, Washington University, St. Louis, MO USA; ^3^ NeuroGenomics & Informatics Center, Washington University School of Medicine, St. Louis, MO USA; ^4^ Department of Psychiatry, Washington University School of Medicine, St. Louis, MO USA

## Abstract

**Background:**

While several non‐invasive plasma biomarkers are rapidly developing, they still have lower accuracy than the established CSF biomarkers. This study therefore comprehensively examined 6,905 plasma proteins in over 3,000 individuals to identify novel biomarkers for predicting clinical Alzheimer’s disease (AD) and monitoring disease progression.

**Method:**

To identify and validate plasma biomarkers, we performed difference abundance analysis of 6,905 plasma proteins in a total of 3,366 cases and controls from three different datasets (Knight‐ADRC discovery, Knight‐ADRC replication and Stanford ADRC). Enrichment analysis with Gene Ontology and DisGeNet were performed to understand biological significance of the identified proteins. Machine learning model was used to create prediction models for AD risk, which were replicated on the Knight ADRC replication and Stanford datasets.

**Result:**

We identified and replicated 257 plasma proteins (including NEFL, SMOC1, SPON1, and NEUROG1) associated with clinical AD status. Machine learning model identified 89 proteins with strong prediction power for AD (AUC=0.843 in Knight ADRC; AUC=0.771 in Stanford ADRC), but not for Parkinson's disease (AUC< 0.6 in two independent datasets), frontotemporal dementia (AUC=0.726), and dementia with Lewy bodies (AUC=0.712), indicating AD specificity. This proteomic signature also predicted AD individuals with faster progression (clinical dementia rating sum‐of‐boxes changes per year; P=4.7∗10^‐5^). Furthermore, this model identified a subset of cognitive normal individuals at 201 times higher risk of developing AD individuals (hazard ratio =201.3; P=1.95∗10^‐2^). Pathway analysis highlighted proteins (APOE, SPP1, and PLTP) related to lipid transport pathways (FDR=2.7∗10^‐2^). DisGeNet indicated the relevance to senile cardiac amyloidosis (APOE, CRP, and NEFL; FDR=1.1∗10^‐4^), highlighting transthyretin, that was found to be potentially protective against amyloid beta deposition.

**Conclusion:**

This large‐scale plasma proteomic study identified proteins associated with AD, developed a robust prediction model that accurately predicted AD status, examined differences between predicted groups in progression and conversion, and revealed pathways and diseases relevant to AD. These findings from extensive analysis provides the potential of plasma proteins as biomarkers for routine clinical uses in early detection of AD and guiding AD treatment decisions. They provide valuable insights into the relevance of specific pathways and diseases in the context of AD.

